# Heteronuclear transverse and longitudinal relaxation in AX_4_ spin systems: Application to ^15^N relaxations in ^15^NH_4_^+^

**DOI:** 10.1016/j.jmr.2014.06.010

**Published:** 2014-09

**Authors:** Nicolas D. Werbeck, D. Flemming Hansen

**Affiliations:** Institute of Structural and Molecular Biology, Division of Biosciences, University College London, London WC1E 6BT, United Kingdom

**Keywords:** AX_4_ spin systems, Nuclear spin relaxation, Ammonium

## Abstract

The equations that describe the time-evolution of transverse and longitudinal ^15^N magnetisations in tetrahedral ammonium ions, ^15^NH_4_^+^, are derived from the Bloch-Wangsness-Redfield density operator relaxation theory. It is assumed that the relaxation of the spin-states is dominated by (1) the intra-molecular ^15^N–^1^H and ^1^H–^1^H dipole–dipole interactions and (2) interactions of the ammonium protons with remote spins, which also include the contribution to the relaxations that arise from the exchange of the ammonium protons with the bulk solvent. The dipole–dipole cross-correlated relaxation mechanisms between each of the ^15^N–^1^H and ^1^H–^1^H interactions are explicitly taken into account in the derivations. An application to ^15^N-ammonium bound to a 41 kDa domain of the protein DnaK is presented, where a comparison between experiments and simulations show that the ammonium ion rotates rapidly within its binding site with a local correlation time shorter than approximately 1 ns. The theoretical framework provided here forms the basis for further investigations of dynamics of AX_4_ spin systems, with ammonium ions in solution and bound to proteins of particular interest.

## Highlights

• Group theory is used to simplify the calculations of nuclear spin relaxation rates.
• Transverse and longitudinal ^15^N nuclear relaxation rates in ^15^NH_4_^+^ are derived.
• An ammonium ion bound to a protein domain rotates rapidly within its binding site.

## Graphical abstract

**Figure:**
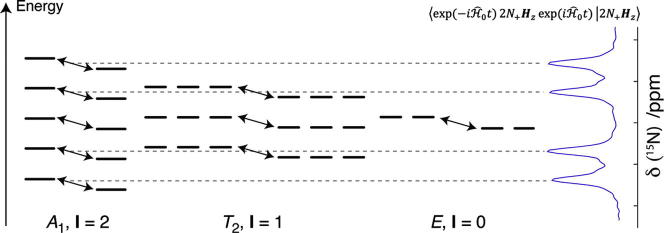

## 1 Introduction

The transverse and longitudinal nuclear spin-relaxation rates, which can be obtained from NMR spectra, are accurate reporters on the interactions and dynamics of molecules ranging from small organic molecules and ions [1], [2], [3], [4] to large macromolecular complexes [5], [6], [7], [8]. The observed relaxation rates can be modulated when the nuclei in question exchange between different magnetic environments, which has stimulated the development of theory [9] and solution-state NMR pulse sequences [10], [11], [12] to probe chemical exchange from nuclear relaxation rates and also methods to separate the contributions from exchange and internal dynamics [13], [14].

Under physiological conditions, the chemical exchange of the ^15^NH_4_^+^ protons with the bulk solvent is so fast that these protons are barely observed in even simple one-dimensional ^1^H NMR spectra. Moreover, the exchange rate of the ammonium protons with the bulk solvent is often much faster than the ^15^N–^1^H scalar coupling [15] thus hindering the acquisition of two-dimensional ^15^N–^1^H correlation spectra. However, under certain conditions, including acidic aqueous solutions and when the ammonium ion is bound to proteins [16] or nucleic acid complexes [17], [18], [19], the exchange rate of the ammonium protons becomes sufficiently slow to allow for both detection of the ammonium protons and acquisition of ^15^N–^1^H correlation spectra. The feasibility of obtaining such ^15^N–^1^H correlation maps provides a promising tool for characterising the dynamics of the ammonium ion and for correlating the dynamics with the environments.

The ionic radius of the ammonium ion (1.44 Å) is similar to the radius of the potassium ion (1.33 Å), so that ammonium can be used as a proxy for potassium to probe potassium binding sites [16], [17], [18], [19] in proteins and nucleic acids. As was shown recently [16], ^15^NH_4_^+^ can be observed even when bound to proteins with molecular weights in excess of 40 kDa, but it is currently not clear whether it is fast reorientation of the ammonium ion within the binding site or favourable cross-correlated relaxation mechanisms that allow for such measurements.

Given the development of techniques to probe ammonium ions in proteins and nucleic acids and also considering the interest in probing the regulations of enzymes by monovalent cations in general, it is of interest to derive equations that describe the transverse and longitudinal relaxations of ammonium ions under various conditions. A derivation of the ^15^N relaxation rates of ammonium ions is presented here, which is based on Bloch-Wangsness-Redfield relaxation theory as well as group theory. An application to ^15^N-ammonium bound to a 41 kDa domain of the protein DnaK is presented, illustrating the utility of the derived equations.

## 2 Theory and results

The longitudinal and transverse relaxation rates of the various spin-states of the ^15^N-ammonium AX_4_ spin-system are calculated using the Bloch-Wangsness-Redfield theory [20], [21], [22], [23]. We assume here that the geometric structure of the AX_4_ spin-system is that of a tetrahedron, which for ammonium means that the ^15^N nucleus is in the centre, with each of the four protons located at the corners of the tetrahedron (see below). Thus, the symmetry-adapted elements of an irreducible basis representation have symmetries that fall within the irreducible representations of the *T_d_* point group [24], that is, *A*_1_, *A*_2_, *E*, *T*_1_, *T*_2_. The total spin density operator that completely describes the spin-state of ^15^NH_4_^+^ can be written as a direct product of spin density operators describing the ^15^N and proton spin-states. The ^15^N and proton spin density operators can in turn be expressed as linear combinations of a set of basis operators. Here we derive ^15^N relaxation rates in terms of two sets of proton spin density basis operators: (1) Proton spin density operators that are the projection operators of the eigenfunctions to the proton Zeeman Hamiltonian. These energy eigenfunctions are denote by |*m*_1_ *m*_2_ *m*_3_ *m*_4_〉, where *m_i_* (*i* = 1, 2, 3, 4) is the eigenvalue of the Zeeman Hamiltonian (*α* ≡ 1/2, *β* ≡ −1/2). The corresponding projection operator, which is the relevant density operator element, is denoted by |*m*_1_ *m*_2_ *m*_3_ *m*_4_〉〈*m*_1_ *m*_2_ *m*_3_ *m*_4_|. (2) Proton spin density operators from the basis of Cartesian/shift operator basis, where each basis operator represents a combination of longitudinal and zero-quantum magnetisations of the four protons, that is, {*H*_z1_, *H*_z2_, … , *H*_z1_ *H*_z2_, … , *H*_+1_ *H*_−2_, … , *H*_z1_ *H*_z2_ *H*_z3_ *H*_z4_}, where *H*_z_ *_i_* is the longitudinal product operator of proton *i*, and *H*_+_ *_i_* and *H*_−_ *_i_* are the corresponding shift (raising and lowering) operators. As shown below, we use group theory to derive symmetry-adapted proton spin eigenfunctions, thereby simplifying the calculation of the relaxation rates.

### 2.1 Constructing symmetry-adapted basis functions

Symmetry-adapted basis functions for the spin wavefunctions in tetrahedral *T*_d_ symmetry can be conveniently constructed with the basic tools of group theory. The major strength of using the symmetry-adapted basis functions, as opposed to non-symmetry adapted functions, is that time-evolutions are simpler since total-symmetric Hamiltonians (*A*_1_ in the *T*_d_ point group) cannot mix functions with different symmetry. In the context of NMR spectroscopic investigations of AX_4_ spin-systems, this means that the time-evolution of the spin-system and the observed relaxation rates are more intuitive. Below we briefly outline how the symmetry-adapted basis functions, which are also eigenfunctions of the proton Zeeman Hamiltonian, H^Z, are constructed.

For the four X spins of the AX_4_ spin system, for example the four protons of the ammonium ion, the Zeeman basis consists of 16 elements, which we denote as {|αααα〉, |αααβ〉, |ααβα〉, ... , |ββββ〉}, and which satisfy the following eigenvalue equation:

(1) H^Z|m1m2m3m4〉=(m1+m2+m3+m4)ℏωH|m1m2m3m4〉

The symmetry-operations within the *T*_d_ point group are those of one *E* (identity operator), eight *C*_3_ axes (proper rotations), three *C*_2_ axes (proper rotations), six *S*_4_ axes (improper rotations), and six *σ*_d_ planes (dihedral symmetry planes) [24]. Thus, the order of the *T*_d_ group, *h*, is 24, and the *T*_d_ point-group is isomorphic to the *S*_4_ symmetric group of permutations of four elements.

It is noted that the 24 symmetry operations cannot mix states with different eigenvalues to the Zeeman Hamiltonian; that is, the matrix representations of the symmetry elements are block-diagonal. The function |αααα〉 is the only function with eigenvalue $+2\hbar \omega_{\text{H}}$ and since this function is total-symmetric it is already an irreducible representation with symmetry *A*_1_. The four functions {|αααβ〉, |ααβα〉, |αβαα〉, |βααα〉} are the only functions with eigenvalue of $+\hbar \omega_{\text{H}}$ and these functions are therefore considered separately. The number of symmetry-adapted basis functions within each of the irreducible representations of the *T_d_* group is determined using Schur’s orthogonality theorems [24], [25] that leads to

(2) $$a_{l}=\frac{1}{h}\underset{c}{\sum }g(c)\chi^{\left(l\right)}{\left(c\right)}^{*}\chi (c)$$

where *a_l_* is the number of functions with representation *l*, the sum is over the classes *c* of symmetry operations, *g*(*c*) is the number of operations within the class, and $\chi^{\left(l\right)}(c)$ and χ(c) are the characters of the representation *l* and of the set of functions in question, respectively. The characters $\chi^{\left(l\right)}(c)$ are available from standard character-tables while χ(c) is simply the number of basis functions that do not change under the given symmetry operation. Thus,

(3) $$a_{A1}=\frac{1}{24}(1\times 1\times 4+8\times 1\times 1+3\times 1\times 0+6\times 1\times 0+6\times 1\times 2)=1$$

(4) $$a_{A2}=\frac{1}{24}(1\times 1\times 4+8\times 1\times 1+3\times 1\times 0+6\times (-1)\times 0+6\times (-1)\times 2)=0$$

(5) $$a_{E}=a_{T1}=0$$

(6) $$a_{T2}=\frac{1}{24}(1\times 3\times 4+8\times 0\times 1+3\times (-1)\times 0+6\times (-1)\times 0+6\times 1\times 2)=1$$

The four basis functions, {|αααβ〉, |ααβα〉, |αβαα〉, |βααα〉}, therefore span one function with *A*_1_ symmetry and three functions with *T*_2_ symmetry (the order of the *T*_2_ symmetry is three). The full set of symmetry-adapted functions are now generated from the original set by applying the 24 symmetry operations and multiplying by the character of the symmetry operation in question as detailed elsewhere [24], [25]. Thus, generation from |αααβ〉 gives,(7)


**Fig. 1:**
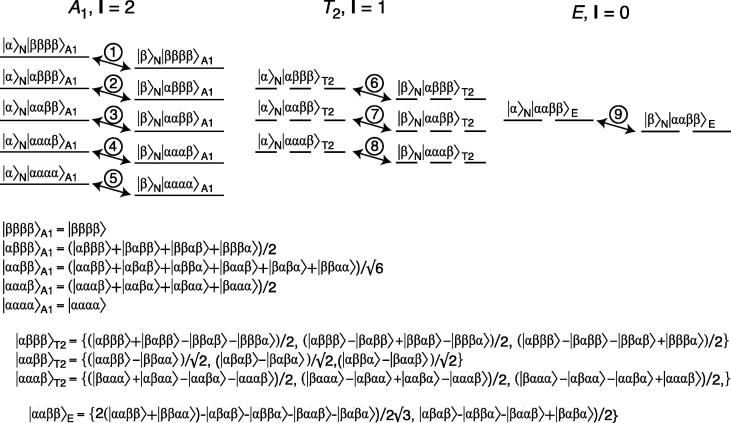
Energy level diagram and nitrogen transitions within the Zeeman basis for the AX_4_ spin-system, exemplified by the ^15^NH_4_^+^ ammonium ion that forms the basis for the theoretical framework and relaxation rate calculations presented here. Shown from the top-left are a spin-2 manifold with *A*_1_ symmetry, three degenerate spin-1 manifolds with *T*_2_ symmetry and two degenerate spin-0 manifolds (singlet) with *E* symmetry. The ^15^N single-quantum transitions are marked with arrows and numbered.

Three additional functions with *T*_2_ symmetry can be constructed in a similar manner by applying the procedure detailed in Eq. (7) to the other three functions that have an eigenvalue of $+\hbar \omega_{\text{H}}$, that is |ααβα〉, |αβαα〉 and |βααα〉. Finally, a basis set of functions with *T*_2_ symmetry, which consists of three orthonormal functions, can be constructed from linear combinations of the four functions generated above. Although the exact form of such a basis set can vary, we chose here to use the three functions that are also eigenfunctions to the *C*_2_ operators as basis functions; these functions are given in Fig. 1 . The linear combination with *A*_1_ symmetry can be generated following a strategy similar to the one given above, yielding:

(8) $${|\alpha \alpha \alpha \beta \rangle }_{A1}=(|\alpha \alpha \alpha \beta \rangle +|\alpha \alpha \beta \alpha \rangle +|\alpha \beta \alpha \alpha \rangle +|\beta \alpha \alpha \alpha \rangle )/2$$

Following the method outlined above in Eqs. (1), (2), (3), (4), (5), (6), the six basis functions with eigenvalue of 0 to the proton Zeeman Hamiltonian, {|ααββ〉, ... , |ββαα〉}, can be shown to span one function with *A*_1_ symmetry, three functions with *T*_2_ symmetry and two functions with *E* symmetry. The function with *A*_1_ symmetry is trivially given by the sum of the six elements:

(9) $${|\alpha \alpha \beta \beta \rangle }_{A1}=(|\alpha \alpha \beta \beta \rangle +|\alpha \beta \alpha \beta \rangle +|\alpha \beta \beta \alpha \rangle +|\beta \alpha \alpha \beta \rangle +|\beta \alpha \beta \alpha \rangle +|\beta \beta \alpha \alpha \rangle )/\sqrt{6}$$

The functions with *T*_2_ symmetry and *E* symmetry can be generated using the basis function |ααββ〉 for generation and the method outlined in Eq. (7), which gives:

(10) $${|\alpha \alpha \beta \beta \rangle }_{T2}=(|\alpha \alpha \beta \beta \rangle -|\beta \beta \alpha \alpha \rangle )/\sqrt{2}$$

(11) $${|\alpha \alpha \beta \beta \rangle }_{E}=(2|\alpha \alpha \beta \beta \rangle -|\alpha \beta \alpha \beta \rangle -|\alpha \beta \beta \alpha \rangle -|\beta \alpha \alpha \beta \rangle -|\beta \alpha \beta \alpha \rangle +2|\beta \beta \alpha \alpha \rangle )/2\sqrt{3}$$

The function given in Eq. (10), along with the other functions with *T*_2_ symmetry that are directly generated following the method described above, are already eigenfunctions to the *C*_2_ operators. The full set of three orthonormal basis functions is given in Fig. 1. Moreover, the function given in Eq. (11) with *E* symmetry is also already an eigenfunction to the *C*_2_ operators. Finally, the symmetry-adapted functions, |αβββ〉_A1_, |αβββ〉_T2_, |ββββ〉_A1_, are obtained by exchanging α for β and β for α in the functions obtained above, i.e., |αααβ〉_A1_, |αααβ〉_T2_, |αααα〉_A1_. The resulting energy level diagram and the orthonormal basis functions are shown in Fig. 1, which also shows the nitrogen transitions coupled to the Zeeman symmetry-adapted basis set of proton spin-states.

### 2.2 Transitions and heteronuclear transverse relaxation within the *AX*_4_ spin-system

Fig. 1 shows the symmetry-adapted basis functions for the Zeeman Hamiltonian in the tetrahedral ammonium ion. An important consequence of the tetrahedral symmetry of the ammonium ion is that a total-symmetric Hamiltonian, which is invariant under the symmetry operations of the molecule, can only mix states with the same symmetry. Therefore, the five eigenfunctions with *A*_1_ symmetry, {|αααα〉_A1_, |αααβ〉_A1_, |ααββ〉_A1_, |αβββ〉_A1_, |ββββ〉_A1_}, form a separate spin-2 manifold; the functions with *T*_2_ symmetry form a degenerate set of three spin-1 manifolds, while the functions with *E* symmetry form two spin-0 manifolds (singlets).

The angular frequencies of the nine nitrogen transitions shown in Fig. 1 depend both on the total Zeeman Hamiltonian, H^Z=(Hz1+Hz2+Hz3+Hz4)ωH+NzωN and the ^15^N–^1^H scalar-coupling Hamiltonian, H^J=πJNH(2Hz1Nz+2Hz2Nz+2Hz3Nz+2Hz4Nz). The transitions ν_1_ = *N*_+_(|ββββ〉〈ββββ|_A1_) and ν_5_ = *N*_+_(|αααα〉〈αααα|_A1_) therefore form the two outer-most lines of the AX_4_ quintet, the central line is formed from ν_3_, ν_7_ and ν_9_ and {ν_2_, ν_6_} and {ν_4_, ν_8_} form the remaining two lines. Here the transitions associated with a degenerate set of manifolds are constructed as normalised sums, as described previously [26], for example, ν_6_ = *N*_+_(|αβββ〉〈αβββ|_T2,1_ + |αβββ〉〈αβββ|_T2,2_ + |αβββ〉〈αβββ|_T2,3_)$/\sqrt{3}$, where |αβββ〉〈αβββ|_T2,i_ symbolise the population operators of the three degenerate levels (*i* = 1, 2, 3) within the *T*_2_ symmetry.

When transverse ^15^N magnetisation of the ammonium ion is created in a standard NMR experiment the spin-state is conveniently described using the product operator formalism [27]. Here, the equilibrium density operator, *σ*_eq,_ of the spin system can be written: *σ*_eq_ ∝ *γ*_H_ (*H*_z1_ + *H*_z2_ + *H*_z3_ + *H*_z4_) + *γ*_N_ *N*_z_, where *γ*_H_ and *γ*_N_ are the gyromagnetic ratios of the proton and the nitrogen, respectively, and *H*_z1_, … , *H*_z4_ and *N*_z_ are the canonical Cartesian product operator density elements describing the longitudinal magnetisations of the four protons and the nitrogen spin, respectively. The equilibrium density operator, *σ*_eq_, contains the sum of the longitudinal magnetisation of all the protons and the symmetry of *σ*_eq_ is therefore totally-symmetric *A*_1_ representation. Density operators created by evolving the ^1^H–^15^N scalar coupling Hamiltonian will therefore also be of *A*_1_ symmetry. For example, the first INEPT of a standard ^1^H–^15^N correlation experiment, 90_x_(^1^H) − 1/4*J*_NH_ − 180_x_(^1^H,^15^N) − 1/4*J*_NH_ − 90_y_(^1^H), will lead to a density operator proportional to 2*N*_z_(*H*_z1_ + *H*_z2_ + *H*_z3_ + *H*_z4_), which we denote 2*N*_z_ ***H***_z_. For calculations of time-evolutions of the AX_4_ spin-system it is therefore also often convenient to consider the basis constructed from the Cartesian operators; Table 1 provides the relationship between the two basis sets in the context of transverse ^15^N magnetisation for the ammonium ion.

**Table 1:** Relationship between the elements of the Cartesian longitudinal product operator basis and the transitions of the Zeeman basis.

Cartesian basis^a^	Zeeman basis
*N*_+_	$\nu_{1}+\nu_{2}+\nu_{3}+\nu_{4}+\nu_{5}+\sqrt{3}\nu_{6}+\sqrt{3}\nu_{7}+\sqrt{3}\nu_{8}+\sqrt{2}\nu_{9}$
2*N*_+_***H***_z_	$4\nu_{1}+2\nu_{2}+0\nu_{3}-2\nu_{4}-4\nu_{5}+2\sqrt{3}\nu_{6}+0\nu_{7}-2\sqrt{3}\nu_{8}+0\nu_{9}$
4*N*_+_***H***_z_***H***_z_	$6\nu_{1}+0\nu_{2}-2\nu_{3}+0\nu_{4}+6\nu_{5}-0\nu_{6}-2\sqrt{3}\nu_{7}+0\nu_{8}+2\sqrt{2}\nu_{9}$
8*N*_+_***H***_z_***H***_z_***H***_z_	$4\nu_{1}-2\nu_{2}+0\nu_{3}+2\nu_{4}-4\nu_{5}-2\sqrt{3}\nu_{6}+0\nu_{7}+2\sqrt{3}\nu_{8}+0\nu_{9}$
16*N*_+_***H***_z_***H***_z_***H***_z_***H***_z_	$\nu_{1}-\nu_{2}+\nu_{3}-\nu_{4}+\nu_{5}-\sqrt{3}\nu_{6}+\sqrt{3}\nu_{7}-\sqrt{3}\nu_{8}+\sqrt{2}\nu_{9}$
*N*_+_***H***_+_***H***_−_	$0\nu_{1}+3\nu_{2}+4\nu_{3}+3\nu_{4}+0\nu_{5}-\sqrt{3}\nu_{6}+0\nu_{7}-\sqrt{3}\nu_{8}-2\sqrt{2}\nu_{9}$
2*N*_+_***H***_+_***H***_−_***H***_z_	$0\nu_{1}+6\nu_{2}+0\nu_{3}-6\nu_{4}+0\nu_{5}-2\sqrt{3}\nu_{6}+0\nu_{7}+2\sqrt{3}\nu_{8}+0\nu_{9}$
4*N*_+_***H***_+_***H***_−_***H***_z_***H***_z_	$0\nu_{1}+3\nu_{2}-4\nu_{3}+3\nu_{4}+0\nu_{5}-\sqrt{3}\nu_{6}+0\nu_{7}-\sqrt{3}\nu_{8}+2\sqrt{2}\nu_{9}$
*N*_+_***H***_+_***H***_−_***H***_+_***H***_−_	$0\nu_{1}+0\nu_{2}+4\nu_{3}+0\nu_{4}+0\nu_{5}+0\nu_{6}-4\sqrt{3}\nu_{7}-0\nu_{8}+4\sqrt{2}\nu_{9}$

a The following notation has been used: ***H***_z_ *=* *H*_z1_ + *H*_z2_ + *H*_z3_ + *H*_z4_; ***H***_z_***H***_z_ *=* *H*_z1_*H*_z2_ + *H*_z1_*H*_z3_ + *H*_z1_*H*_z4_ + *H*_z2_*H*_z3_ + *H*_z2_*H*_z4_ + *H*_z3_*H*_z4_; ***H***_z_***H***_z_***H***_z_ = *H*_z1_*H*_z2_*H*_z3_ + *H*_z1_*H*_z2_*H*_z4_ + *H*_z1_*H*_z3_*H*_z4_ + *H*_z2_*H*_z3_*H*_z4_; ***H***_z_***H***_z_***H***_z_***H***_z_ *=* *H*_z1_*H*_z2_*H*_z3_*H*_z4_; ***H***_+_***H***_−_ = $\sum_{i\neq j}H_{+\text{,}i}H_{-\text{,}j}$; ***H***_+_***H***_−_***H***_z_ *=* $\sum_{i\neq j\neq k}H_{+\text{,}i}H_{-\text{,}j}H_{z\text{,}k}$; ***H***_+_***H***_−_***H***_z_***H***_z_ *=* $\sum_{i\neq j\neq k\neq l}H_{+\text{,}i}H_{-\text{,}j}H_{z\text{,}k}H_{z\text{,}l}$; ***H***_+_***H***_−_***H***_+_***H***_−_ *=* $\sum_{i\neq j\neq k\neq l}H_{+\text{,}i}H_{-\text{,}j}H_{+\text{,}k}H_{-\text{,}l}$.

### 2.3 Time-evolution of the spin-system

**Fig. 2:**
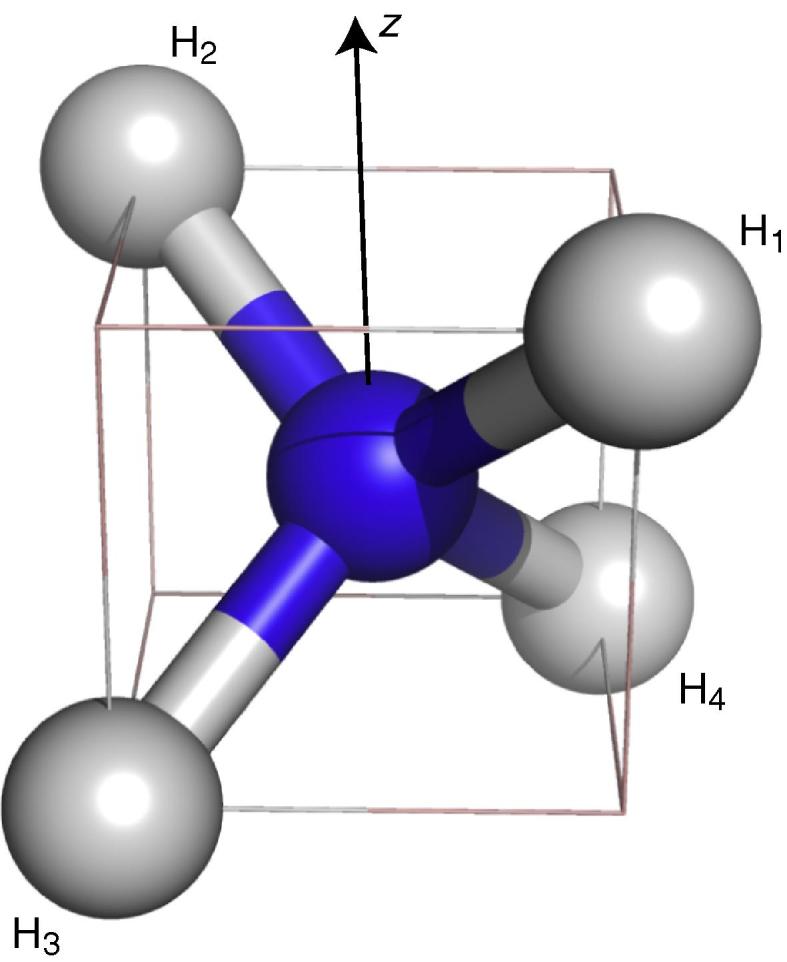
A schematic representation of the molecular coordinate frame used here to calculate the nitrogen relaxation rates. The nitrogen atom (blue) is placed at the origin, the proton H_1_ at $\left(r_{\text{NH}}/\sqrt{3}\right)${1, 1, 1}, H_2_ at $\left(r_{\text{NH}}/\sqrt{3}\right)${−1, −1, 1}, H_3_ at $\left(r_{\text{NH}}/\sqrt{3}\right)${1, −1, −1}, and H_4_ at $\left(r_{\text{NH}}/\sqrt{3}\right)${−1, 1, −1}. The spherical coordinates of the interaction vectors were calculated based on the positions shown above, for example, for the ^15^N–^1^H_1_ interaction the spherical coordinates $\left(\Omega_{\text{N}\text{"–"}\text{H}1}^{\text{mol}}\right)$ are $\theta =\cos^{-1}\frac{1}{\sqrt{3}}\text{,}\;\phi =\pi /4$.

Following the Bloch-Wangsness-Redfield theory [20], [21], [22], [23], the evolution of the spin-system is given by the Liouville-von Neumann equation,

(12) dσ(t)dt=-i[H^0,σ(t)]-Γ^(σ(t)-σeq)

where H^0 is the time-independent part of the Hamiltonian, *σ*_eq_ is the equilibrium density operator, and Γ^ is the relaxation super-operator, which is derived from the stochastic time-dependent Hamiltonian, H^1(t). The Hamiltonian H^1(t) can be factored into second-rank tensor spin operators and functions that depend on the spatial variables,

(13) H^1(t)=∑m∑q=-22Fm2q(t)Am2q

where the index *m* is over the various interactions, for example, the ^15^N–^1^H_1_ or ^1^H_1_–^1^H_2_ dipole interactions. The time-dependent Hamiltonian can be factorised, such that the functions $F_{\mathrm{mk}}^{q}(t)$, which give the spatial part, are proportional to the spherical harmonic functions, $F_{\mathrm{mk}}^{q}(t)\propto Y_{k}^{q}(\Omega_{m}^{\text{lab}}(t))$, and the tensor spin operators, $A_{m2}^{q}$, are given by the traditional set, as discussed elsewhere [20], [21], [22]. The spherical angle $\Omega_{m}^{\text{lab}}(t)$ is the angle of the interaction-vector of *m* in the laboratory-frame; for the ^15^N–^1^H_1_ interaction this interaction-vector is the ^15^N–^1^H internuclear vector. We will here relate the angle $\Omega_{m}^{\text{lab}}(t)$, of the interaction-vector in the laboratory-frame via a molecular coordinate-frame for the ammonium ion. By doing so, each interaction *m* will then relate to the laboratory frame by a *time-independent* rotation to the molecular frame, $\Omega_{m}^{\text{mol}}$, followed by a time-dependent *interaction–independent* rotation into the laboratory frame. The molecular coordinate frame used here for these rotations for the ammonium ion is shown in Fig. 2 .

Since the functions $F_{\mathrm{mk}}^{q}(t)$ are proportional to the spherical harmonics, $Y_{2}^{q}$, their rotations are governed by the Wigner rotation matrices [28], [29]. The stochastic Hamiltonian can therefore be expressed as:

(14) H^1(t)=∑m∑q=-22∑q′=-22Dq,q′(2)(Ωmmol)FMol,2q′(t)Am2q

where $F_{\mathrm{Mol}\text{,}2}^{q^{\prime }}$ are the random functions that describe the spatial coordinates of the molecular coordinate frame; these functions are independent of the interaction *m*. The relaxation super-operator then becomes:

(15) Γ^=15∑m,n,p,qjm,nq(ωp)[Am2p-q,[An2pq,]]

where $A_{m2p}^{q}$ is the *q* component of the second-rank tensor spin operator for the interaction *m*, with frequency *ω_p_*, and $j_{m\text{,}n}^{q}(\omega_{p})$ is the *q* component of the spectral density function arising from the *m* and *n* interactions, which is calculated from the random functions of spatial variables:

(16) $$j_{m\text{,}n}^{q}(\omega )=\frac{5}{2}\text{Re}\left[\int_{-\infty }^{\infty }d\tau \langle \underset{q^{\prime }}{\sum }D_{q\text{,}q^{\prime }}^{\left(2\right)}(\Omega_{m}^{\text{mol}})F_{\mathrm{Mol}\text{,}2}^{q^{\prime }}(t)\underset{q^{\prime \prime }}{\sum }D_{-q\text{,}q^{\prime \prime }}^{\left(2\right)}(\Omega_{n}^{\text{mol}})F_{\mathrm{Mol}\text{,}2}^{q^{\prime \prime }}(t+\tau )\rangle \exp(-i\omega \tau )\right]$$

Finally, the matrix representation of Γ^ in a basis set B is given by:

(17) Γ^rs=〈Br|Γ^|Bs〉=15∑m,n,p,qjm,nq(ωp)Br[Am2p-q,[An2pq,Bs]]/〈Br|Br〉

For the dipolar I–S interaction we have $F_{\mathrm{Mol}\text{,}2}^{q}(t)=-\sqrt{6}d_{\mathrm{IS}}Y_{2}^{q}(\Omega^{\text{lab}}(t))$, where $d_{\mathrm{IS}}=(\frac{\mu_{0}}{4\pi })\hbar \gamma_{I}\gamma_{S}r_{\mathrm{IS}}^{-3}$ and $\Omega^{\text{lab}}(t)$ is the orientation of the molecular coordinate-frame relative to the laboratory frame. Assuming isotropic tumbling for the symmetric AX_4_ molecule gives [21], [22]:

(18) $$\text{Re}\left[\int_{-\infty }^{\infty }d\tau \langle F_{\mathrm{Mol}\text{,}2}^{q^{\prime }}(t)F_{\mathrm{Mol}\text{,}2}^{q^{\prime \prime }}(t+\tau )\rangle \exp(-i\omega \tau )\right]=\delta_{q^{\prime }\text{,}q^{\prime \prime }}{\left(-1\right)}^{q^{\prime }}\frac{2}{5}\frac{\tau_{c}}{1+\omega^{2}\tau_{c}^{2}}$$

where *τ_c_* is the rotational correlation time of the molecule.

Table 2 summarises the angular frequencies and transverse relaxation rates of spin A for the AX_4_ spin system in the basis set consisting of the transitions between Zeeman levels, exemplified by the relaxation rates of the ammonium ion. The calculations of the relaxation rates include the four ^15^N–^1^H dipolar interactions and the six ^1^H–^1^H dipolar interactions. The chemical shift anisotropy of the ^15^N nucleus is not included here because the chemical shift tensor will be isotropic due to the tetrahedral geometry. For a distorted tetrahedral geometry, for example for an ammonium ion in an anisotropic environment, contributions from chemical shift anisotropy can occur.

**Table 2:** Angular frequencies and transverse heteronuclear relaxation^a^ rates of ^15^N in ammonium ions for the symmetry-adapted Zeeman basis (Fig. 1):

Time evolution	dν¯dt=(-R^+iωZ)ν¯
Angular frequencies	*ω*_11_ = *ω*_N_ − 4*πJ*_NH_
	*ω*_22_ = *ω*_66_ = *ω*_N_ − 2*πJ*_NH_
	*ω*_33_ = *ω*_77_ = *ω*_99_ = *ω*_N_
	*ω*_44_ = *ω*_88_ = *ω*_N_ + 2*πJ*_NH_
	*ω*_55_ = *ω*_N_ + 4*πJ*_NH_
	*ω_ij_* = 0 for *i* ≠ *j*

Relaxation rates	R^1,1=15dNH2(3J(ωH)+J(ωH-ωN)+6J(ωH+ωN))+120dHH2(27J(ωH)+72J(2ωH))+2λ
	R^2,2=110dNH2(4J(0)+3J(ωH)+J(ωH-ωN)+6J(ωN)+6J(ωH+ωN))+940dHH2(9J(0)+7J(ωH)+6J(2ωH))+54λ+32θ
	R^3,3=115dNH2(8J(0)+3J(ωH)+J(ωH-ωN)+12J(ωN)+6J(ωH+ωN))+920dHH2(2J(0)+9J(ωH))+λ+2θ
	R^4,4=R^2,2
	R^5,5=R^1,1
	R^66=130dNH2(20J(0)+21J(ωH)+7J(ωH-ωN)+18J(ωN)+42J(ωH+ωN))+340dHH2(9J(0)+25J(ωH)+26J(2ωH))+74λ+12θ
	R^77=115dNH2(8J(0)+9J(ωH)+3J(ωH-ωN)+12J(ωN)+18J(ωH+ωN))+320dHH2(13J(ωH)+12J(2ωH))+λ+2θ
	R^88=R^66
	R^99=415dNH2(2J(0)+3J(ωH)+J(ωH-ωN)+3J(ωN)+6J(ωH+ωN))+920dHH2(J(0)+2J(ωH)+2J(2ωH))+λ+2θ
	R^1,2=-12λ
	R^1,3=0
	R^1,4=0
	R^1,5=0
	R^1,6=35dNH2J(ωH)-9320dHH2J(ωH)-32λ
	R^1,7=-9310dHH2J(2ωH)
	R^1,8=0
	R^1,9=-9102dHH2J(2ωH)
	R^2,3=-34λ
	R^2,4=0
	R^2,5=0
	R^2,6=253dNH2J(0)-27340dHH2J(0)+34λ-32θ
	R^2,7=310dNH2J(ωH)-9340dHH2J(ωH)-34λ
	R^2,8=-9320dHH2J(2ωH)
	R^2,9=-9102dHH2J(ωH)
	R^3,4=-34λ
	R^3,5=0
	R^3,6=1103dNH2J(ωH)-27340dHH2J(ωH)-143λ
	R^3,7=8153dNH2J(0)+13λ-23θ
	R^3,8=1103dNH2J(ωH)-27340dHH2J(ωH)-143λ
	R^3,9=-9102dHH2J(0)
	R^4,5=-12λ
	R^4,6=-9320dHH2J(2ωH)
	R^4,7=310dNH2J(ωH)-9340dHH2J(ωH)-34λ
	R^4,8=253dNH2J(0)-27340dHH2J(0)+34λ-32θ
	R^4,9=-9102dHH2J(ωH)
	R^5,6=0
	R^5,7=-9310dHH2J(2ωH)
	R^5,8=35dNH2J(ωH)-9320dHH2J(ωH)-32λ
	R^5,9=-9102dHH2J(2ωH)
	R^6,7=15dNH2J(ωH)-34dHH2J(ωH)-34λ
	R^6,8=-32dHH2J(2ωH)
	R^6,9=2523dNH2J(ωH)-23λ
	R^7,8=15dNH2J(ωH)-34dHH2J(ωH)-34λ
	R^7,9=81523dNH2J(0)+23λ-223θ
	R^8,9=2523dNH2J(ωH)-23λ
	R^i,j=R^j,ifori>j

a $J(\omega )=\tau_{c}/(1+\omega^{2}\tau_{c}^{2})\text{,}$*d*_HH_ and *d*_NH_ are defined in the text, and *λ* and *θ* are the selective longitudinal and transverse relaxation rates, respectively, of the ammonium protons caused by external spins and chemical exchange.

In the spin-1 manifolds with *T*_2_ symmetry, Fig. 1, there are three degenerate states for each eigenvalue of the proton Zeeman Hamiltonian and in the spin-0 singlet manifolds with *E* symmetry there are two degenerate states. Since relaxation is not able to lift these degeneracies, as is also the case for the symmetric states of a rapidly rotating methyl group [30], it is sufficient to calculate the relaxation rates for just one of the degenerate states within each set. For example, the three transitions {*N*_+_|αβββ〉〈αβββ|_T2,1_, *N*_+_|αβββ〉〈αβββ|_T2,2_, *N*_+_|αβββ〉〈αβββ|_T2,3_} between the three *T*_2_ symmetry-adapted energy states are combined into one transition ν_6_ = *N*_+_(|αβββ〉〈αβββ|_T2,1_ + |αβββ〉〈αβββ|_T2,2_ + |αβββ〉〈αβββ|_T2,3_)/$\sqrt{3}$, which is considered for the calculation of relaxation rates.

In the study of macromolecules and large macromolecular complexes it is often of interest to identify spin-states with slow transverse relaxation rates, as for example are explained in the ^15^N–^1^H TROSY [31] or the ^13^CH_3_ methyl-TROSY [32], [33] techniques. For the AX_4_ spin-system, the two outermost lines, *N*_+_|αααα〉〈αααα|_A1_ and *N*_+_|ββββ〉〈ββββ|_A1_, are potential candidates, since their transverse relaxation rates do not depend on the spectral density at zero frequency, *J*(0). This situation arises here because the matrix-representation of the dipolar Hamiltonian is traceless and the four protons, here all with the same spin quantum number, are placed in a symmetric tetrahedron around the nitrogen thus leading to cancellations of the dipolar field at the position of the nitrogen. The cancellation of the dipolar interactions means that the outer ^15^N NMR lines of slow-tumbling ammonium ions can appear significantly sharper than would be expected from only considering the auto-relaxation of the nitrogen nucleus by the four protons. As detailed below, it should be noted that the two outermost lines also relax due to interactions with external spins and chemical exchange with the bulk solvent, thus leading to line-broadening.

It is often convenient to consider the evolution of the spin-system using the basis of Cartesian density spin-operators, for example because the effect of interactions with external spins is diagonal to first approximation [32]. Moreover, those spin operators with *A*_1_ symmetry are of special interest here because these can easily be generated from the equilibrium spin-density operator of the spin-system. Table 3 summarises the angular frequencies and transverse relaxation rates of the Cartesian density spin-operators.

**Table 3:** Angular frequencies and transverse heteronuclear relaxation rates of ^15^N for the spin-operators of the Cartesian basis:

Time evolution	ddtN+2N+Hz4N+HzHz8N+HzHzHz16N+HzHzHzHzN+H+H-2N+H+H-Hz4N+H+H-HzHzN+H+H-H+H-=-R^C+iω^C+λ^ext+θ^extN+2N+Hz4N+HzHz8N+HzHzHz16N+HzHzHzHzN+H+H-2N+H+H-Hz4N+H+H-HzHzN+H+H-H+H-

Angular frequencies	${\overset{ˆ}{\omega }}_{\text{C}}=\pi J_{\mathrm{NH}}\left(\begin{array}{ccccccccc} 0 & 4 & 0 & 0 & 0 & 0 & 0 & 0 & 0\\ 1 & 0 & 3 & 0 & 0 & 0 & 0 & 0 & 0\\ 0 & 2 & 0 & 2 & 0 & 0 & 0 & 0 & 0\\ 0 & 0 & 3 & 0 & 1 & 0 & 0 & 0 & 0\\ 0 & 0 & 0 & 4 & 0 & 0 & 0 & 0 & 0\\ 0 & 0 & 0 & 0 & 0 & 0 & 2 & 0 & 0\\ 0 & 0 & 0 & 0 & 0 & 1 & 0 & 1 & 0\\ 0 & 0 & 0 & 0 & 0 & 0 & 2 & 0 & 0\\ 0 & 0 & 0 & 0 & 0 & 0 & 0 & 0 & 0 \end{array}\right)+\omega_{N}\overset{ˆ}{1}$

Transverse relaxation rates	R^C,11=15dNH2(4J(0)+6J(ωH)+3J(ωN)+J(ωH-ωN)+6J(ωH+ωN))
	R^C,21=0
	R^C,31=-130dNH2(4J(0)+3J(ωN))
	R^C,41=0
	R^C,51=0
	R^C,61=-130dNH2(6J(ωH)+J(ωH-ωN)+6J(ωH+ωN))
	R^C,71=0
	R^C,81=0
	R^C,91=0
	R^C,12=0
	R^C,22110dNH2(4J(0)+9J(ωH)+2J(ωH-ωN)+3J(ωN)+12J(ωH+ωN))+110dHH2(9J(ωH)+36J(2ωH))
	R^C,32=0
	R^C,42=-110dNH2(4J(0)+3J(ωN))-940dHH2(J(ωH))
	R^C,52=0
	R^C,62=0
	R^C,72=-130dNH2(6J(ωH)+J(ωH-ωN)+6J(ωH+ωN))+320dHH2(J(ωH)-2J(2ωH))
	R^C,82=0
	R^C,92=0
	R^C,13=-15dNH2(4J(0)+3J(ωN))
	R^C,23=0
	R^C,33=115dNH2(4J(0)+9J(ωH)+3J(ωH-ωN)+3J(ωN)+18J(ωH+ωN))+310dHH2(5J(ωH)+16J(2ωH))
	R^C,43=0
	R^C,53=-15dNH2(4J(0)+3J(ωN))-910dHH2J(ωH)
	R^C,63=130dNH2(J(ωH-ωN)+6J(ωH+ωN))-320dHH2(J(ωH)-4J(2ωH))
	R^C,73=0
	R^C,83=-130dNH2(6J(ωH)+J(ωH-ωN)+6J(ωH+ωN))+310dHH2(3J(ωH)-2J(2ωH))
	R^C,93=65dHH2J(2ωH)
	R^C,14=0
	R^C,24=-110dNH2(4J(0)+3J(ωN))-940dHH2J(ωH)
	R^C,34=0
	R^C,44=110dNH2(4J(0)+3J(ωH)+2J(ωH-ωN)+3J(ωN)+12J(ωH+ωN))+920dHH2(5J(ωH)+8J(2ωH))
	R^C,54=0
	R^C,64=0
	R^C,74=130dNH2(J(ωH-ωN)+6J(ωH+ωN))+310dHH2(J(ωH)+J(2ωH))
	R^C,84=0
	R^C,94=0
	R^C,15=0
	R^C,25=0
	R^C,35=-130dNH2(4J(0)+3J(ωN))-320dHH2J(ωH)
	R^C,45=0
	R^C,55=15dNH2(4J(0)+J(ωH-ωN)+3J(ωN)+6J(ωH+ωN))+185dHH2J(ωH)
	R^C,65=35dHH2J(ωH)
	R^C,75=0
	R^C,85=130dNH2(J(ωH-ωN)+6J(ωH+ωN))-920dHH2J(ωH)
	R^C,95=0
	R^C,16=-110dNH2(6J(ωH)+J(ωH-ωN)+6J(ωH+ωN))
	R^C,26=0
	R^C,36=160d2(J(ωH-ωN)+6J(ωH+ωN))-340dHH2(J(ωH)-4J(2ωH))
	R^C,46=0
	R^C,56=95dHH2J(ωH)
	R^C,66=130dNH2(12J(0)+15J(ωH)+4J(ωH-ωN)+21J(ωN)+24J(ωH+ωN))+340dHH2(27J(0)+46J(ωH)+14J(2ωH))
	R^C,76=0
	R^C,86=-130dNH2(4J(0)+3J(ωN))+340dHH2(9J(0)-9J(ωH)+10J(2ωH))
	R^C,96=-130dNH2(6J(ωH)+J(ωH-ωN)+6J(ωH+ωN))+320dHH2(3J(ωH)-J(2ωH))
	R^C,17=0
	R^C,27=-120dNH2(6J(ωH)+J(ωH-ωN)+6J(ωH+ωN))+940dHH2(J(ωH)-2J(2ωH))
	R^C,37=0
	R^C,47=120d2(J(ωH-ωN)+6J(ωH+ωN))+920dHH2(J(ωH)+J(2ωH))
	R^C,57=0
	R^C,67=0
	R^C,77=115dNH2(4J(0)+6J(ωH)+2J(ωH-ωN)+9J(ωN)+12J(ωH+ωN))+320dHH2(18J(0)+11J(ωH)+8J(2ωH))
	R^C,87=0
	R^C,97=0
	R^C,18=0
	R^C,28=0
	R^C,38=-160dNH2(6J(ωH)+J(ωH-ωN)+6J(ωH+ωN))+320dHH2(3J(ωH)-2J(2ωH))
	R^C,48=0
	R^C,58=110dNH2(J(ωH-ωN)+6J(ωH+ωN))-2720dHH2J(ωH)
	R^C,68-130dNH2(4J(0)+3J(ωN))+340dHH2(9J(0)-9J(ωH)+10J(2ωH))
	R^C,78=0
	R^C,88=130dNH2(12J(0)+9J(ωH)+4J(ωH-ωN)+21J(ωN)+24J(ωH+ωN))+340dHH2(27J(0)+16J(ωH)+14J(2ωH))
	R^C,98=130dNH2(J(ωH-ωN)+6J(ωH+ωN))-320dHH2(4J(ωH)-J(2ωH))
	R^C,19=0
	R^C,29=0
	R^C,39=65dHH2J(2ωH)
	R^C,49=0
	R^C,59=0
	R^C,69=-115dNH2(6J(ωH)+J(ωH-ωN)+6J(ωH+ωN))+310dHH2(3J(ωH)-J(2ωH))
	R^C,79=0
	R^C,89=115dNH2(J(ωH-ωN)+6J(ωH+ωN))-310dHH2(4J(ωH)-J(2ωH))
	R^C,99=15dNH2(3J(ωH)+J(ωH-ωN)+4J(ωN)+6J(ωH+ωN))+320dHH2(13J(ωH)+8J(2ωH))

Relaxation by external spins	${\overset{ˆ}{\lambda }}_{\text{ext}}=-\lambda \mathrm{diag}(0\text{,}1\text{,}2\text{,}3\text{,}4\text{,}0\text{,}1\text{,}2\text{,}0)$
	${\overset{ˆ}{\theta }}_{\text{ext}}=-\theta \mathrm{diag}(0\text{,}0\text{,}0\text{,}0\text{,}0\text{,}2\text{,}2\text{,}2\text{,}4)$

^a^ $J(\omega )=\tau_{c}/(1+\omega^{2}\tau_{c}^{2})$, *d*_HH_ and *d*_NH_ are defined in the text and $\overset{ˆ}{1}$ is the 9 × 9 identity matrix.

### 2.4 Relaxation caused by external sources

Nuclear spins external to the AX_4_ spin system can cause relaxation of the AX_4_ spin-states in a similar manner to the relaxation of spin-states in the –CH_3_ spin-system by ‘external’ nuclear spins [32], [34]. For the ammonium ion, such relaxations could be caused by protons in the vicinity of the protein-bound ammonium ion or by chemical exchange of the ammonium protons with the bulk solvent. We consider here the scenario where only the proton spins of the ammonium ion are relaxed by external spins, which in the Cartesian basis is described by two diagonal matrix operators [34], [35] (see Table 3), one matrix operator for longitudinal relaxation, ${\overset{ˆ}{\lambda }}_{\text{ext}}$, and one for transverse relaxation, ${\overset{ˆ}{\theta }}_{\text{ext}}$:

(19a) $${\overset{ˆ}{\lambda }}_{\text{ext}}=\lambda \mathrm{diag}(0\text{,}1\text{,}2\text{,}3\text{,}4\text{,}0\text{,}1\text{,}2\text{,}0)$$

(19b) $${\overset{ˆ}{\theta }}_{\text{ext}}=\theta \mathrm{diag}(0\text{,}0\text{,}0\text{,}0\text{,}0\text{,}2\text{,}2\text{,}2\text{,}4)$$

In the Zeeman-derived basis of spin operators, the action of the external spins can be calculated by a basis transformation of Eq. (19a), (19b) into the Zeeman-derived basis using the relations of Table 1, and these are denoted by *λ* and *θ* in Table 2. As seen in Table 2, the effect of the interaction of the ammonium protons with external spins is to transfer magnetisation between adjacent transitions of the Zeeman basis. In the NMR spectrum of the AX_4_ spin-system, the relaxation caused by the external protons is thus manifested as a transfer of magnetisation between adjacent lines of the coupled spectrum, for example between the outermost $\omega_{\text{N}}+4\pi J_{\text{NH}}$ line and the $\omega_{\text{N}}+2\pi J_{\text{NH}}$ line.

### 2.5 Longitudinal relaxation within the AX_4_ spin-system

When probing molecular motions and dynamics from nuclear spin-relaxation rates a, combination of transverse and longitudinal relaxation rates often provide a more accurate picture of the molecular dynamics than either one of the rates alone [36], [37]. We have calculated the longitudinal relaxation rates for the longitudinal operators in the product operator basis, which comprise ten operators, denoted by: {*E*/*2*, ***H***_z_, 2***H***_z_ ***H***_z_, 4***H***_z_ ***H***_z_ ***H***_z_, 8***H***_z_ ***H***_z_ ***H***_z_ ***H***_z_, *N_z_*, 2*N_z_**H***_z_, 4*N_z_**H***_z_ ***H***_z_, 8*N_z_**H***_z_ ***H***_z_ ***H***_z_, 16*N_z_**H***_z_ ***H***_z_ ***H***_z_ ***H***_z_}, where *E* is the identity operator. For simplicity we have ignored the zero-quantum proton coherences since these are only generated via cross-correlated relaxation mechanisms and are normally not populated at the start of the NMR experiment. As for the calculation of the transverse relaxation rates, the four ^15^N–^1^H dipolar interactions and the six ^1^H–^1^H dipolar interactions were all included for the calculations of the longitudinal relaxation rates. The obtained rates are given in Table 4 .

**Table 4:** Longitudinal relaxation rates of the basis operators in the Cartesian basis:

Time evolution	ddtE/2Hz2HzHz4HzHzHz8HzHzHzHzNz2NzHz4NzHzHz8NzHzHzHz16NzHzHzHzHz=-R^+λ^extE/2Hz2HzHz4HzHzHz8HzHzHzHzNz2NzHz4NzHzHz8NzHzHzHz16NzHzHzHzHz

Longitudinal relaxation rates	R^1,i=0fori=1,2,…,10
	R^2,1=-2(R^2,2+γN/γHR^2,6)
	R^3,1=0
	R^4,1=-2(R^4,2+γN/γHR^4,6)
	R^5,1=0
	R^6,1=-2(R^6,2+γN/γHR^6,6)
	R^7,1=0
	R^8,1=-2(R^8,2+γN/γHR^8,6)
	R^9,1=0
	R^10,1=-2(R^10,2+γN/γHR^10,6)
	R^2,2=110dNH2(3J(ωH)+J(ωH-ωN)+6J(ωH+ωN))+910dHH2(J(ωH)+4J(2ωH))
	R^3,2=0
	R^4,2=-940dHH2J(ωH)
	R^5,2=0
	R^6,2=-25dNH2(J(ωH-ωN)-6J(ωH+ωN))
	R^7,2=0
	R^8,2=35dHHdNHJ(ωH)
	R^9,2=0
	R^10,2=0
	R^2,3=0
	R^3,3=15dNH2(3J(ωH)+J(ωH-ωN)+6J(ωH+ωN))+310dHH2(5J(ωH)+16J(2ωH))
	R^4,3=0
	R^5,3=-910dHH2J(ωH)
	R^6,3=0
	R^7,3=-310dNH2(J(ωH-ωN)-6J(ωH+ωN))+910dHHdNHJ(ωH)
	R^8,3=0
	R^9,3=95dHHdNHJ(ωH)
	R^10,3=0
	R^2,4=-940dHH2J(ωH)
	R^3,4=0
	R^4,4=310dNH2(3J(ωH)+J(ωH-ωN)+6J(ωH+ωN))+920dHH2(5J(ωH)+8J(2ωH))
	R^5,4=0
	R^6,4=0
	R^7,4=0
	R^8,4=-15dNH2(J(ωH-ωN)-6J(ωH+ωN))+65dHHdNHJ(ωH)
	R^9,4=0
	R^10,4=185dHHdNHJ(ωH)
	R^2,5=0
	R^3,5=-320dHH2J(ωH)
	R^5,5=25dNH2(3J(ωH)+J(ωH-ωN)+6J(ωH+ωN))+185dHH2J(ωH)
	R^6,5=0
	R^7,5=0
	R^8,5=0
	R^9,5=-110dNH2(J(ωH-ωN)-6J(ωH+ωN))+910dHHdNHJ(ωH)
	R^10,5=0
	R^2,6=-110dNH2(J(ωH-ωN)-6J(ωH+ωN))
	R^3,6=0
	R^4,6=0
	R^5,6=0
	R^6,6=25dNH2(3J(ωN)+J(ωH-ωN)+6J(ωH+ωN))
	R^7,6=0
	R^8,6=-15dNH2J(ωN)
	R^9,6=0
	R^10,6=0
	R^2,7=0
	R^3,7=-15dNH2(J(ωH-ωN)-6J(ωH+ωN))+35dHHdNHJ(ωH)
	R^4,7=0
	R^5,7=0
	R^6,7=0
	R^7,7=310dNH2(J(ωH)+J(ωH-ωN)+2J(ωN)+6J(ωH+ωN))+910dHH2(J(ωH)+4J(2ωH))
	R^8,7=0
	R^9,7=-35dNH2J(ωN)-940dHH2J(ωH)
	R^10,7=0
	R^2,8=910dHHdNHJ(ωH)
	R^3,8=0
	R^4,8=-310dNH2(J(ωH-ωN)-6J(ωH+ωN))+95dHHdNHJ(ωH)
	R^5,8=0
	R^6,8=-65dNH2J(ωN)
	R^7,8=0
	R^8,8=15dNH2(3J(ωH)+J(ωH-ωN)+2J(ωN)+6J(ωH+ωN))+310dHH2(5J(ωH)+16J(2ωH))
	R^9,8=0
	R^10,8=-65dNH2J(ωN)-910dHH2J(ωH)
	R^2,9=0
	R^3,9=65dHHdNHJ(ωH)
	R^4,9=0
	R^5,9=-25dNH2(J(ωH-ωN)-6J(ωH+ωN))+185dHHdNHJ(ωH)
	R^6,9=0
	R^7,9=-35dNH2J(ωN)-940dHH2J(ωH)
	R^8,9=0
	R^9,9=110dNH2(9J(ωH)+J(ωH-ωN)+6J(ωN)+6J(ωH+ωN))+920dHH2(5J(ωH)+8J(2ωH))
	R^10,9=0
	R^2,10=0
	R^3,10=0
	R^4,10=910dHHdNHJ(ωH)
	R^5,10=0
	R^6,10=0
	R^7,10=0
	R^8,10=-15dNH2J(ωN)-320dHH2J(ωH)
	R^9,10=0
	R^10,10=65dNH2(J(ωH)+J(ωN))+185dHH2J(ωH)

Relaxation by external spins	${\overset{ˆ}{\lambda }}_{\text{ext}}=\lambda \;\mathrm{diag}(0\text{,}1\text{,}2\text{,}3\text{,}4\text{,}0\text{,}1\text{,}2\text{,}3\text{,}4)$

### 2.6 Coupled ^15^N spectra of ^15^NH_4_^+^

When the density spin-operator *N*_+_ evolves under the free-precession Hamiltonian and *N*_+_ is directly detected, then a canonical quintet (1:4:6:4:1) reflecting the number and degeneracies of the Zeeman eigenstates (Fig. 1) is observed. When an antiphase coherence is evolved and/or detected, the angular frequencies of the five transitions remain unchanged, but the relative intensities of the NMR lines within the quintet are altered. For example, evolution of the anti-phase coherence 2*N*_+_ ***H***_z_, and detection of *N*_+_ gives a spectrum with relative peak intensities within the quintet of 1:2:0:−2:−1, which can be derived from:

(20) FID(t)=〈exp(-iH^0t)2N+Hzexp(iH^0t)|N+〉

where we have ignored relaxation for the moment. The central line (ν_3_, ν_7_, ν_9_) is not observed since the antiphase coherence 2*N*_+_ ***H***_z_ does not include these transitions (Table 1).

**Fig. 3:**
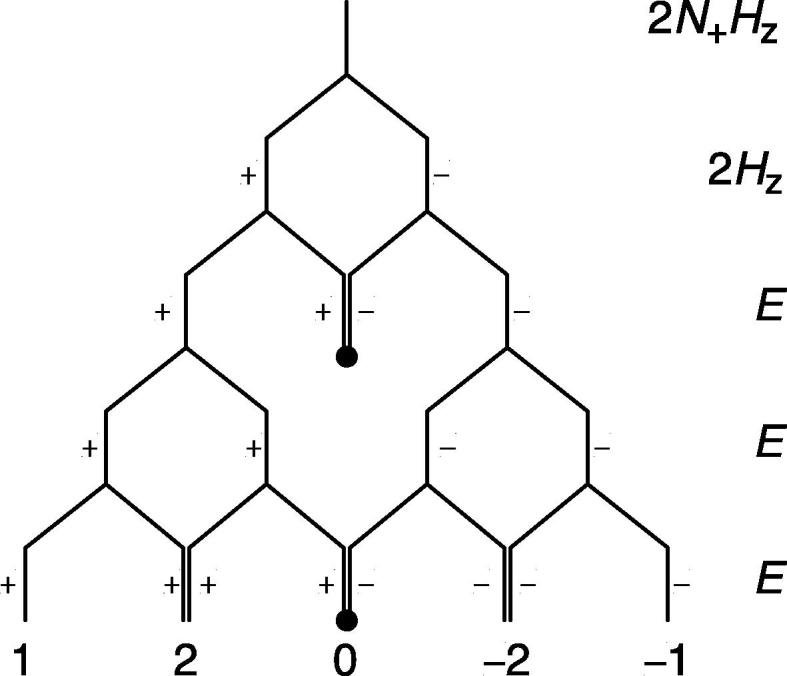
A modified Pascal’s triangle depicting the intuitive derivation of the multiplet structure obtained by evolving/detecting the 2*N*_+_*H*_z_ coherence. The single longitudinal proton density element splits the NMR line in two lines with opposite intensity, while each of the remaining scalar coupled protons splits the NMR line in two lines with equal intensity.

Evolving anti-phase coherences of AX*_n_* spin systems lead to coupling patterns and multiplet structures of the A-spin NMR spectrum that can be intuitively derived from a modified Pascal’s triangle. In the modified Pascal’s triangle presented here, each X spin that is scalar coupled to A and whose spin-state is described with the identity operator splits the NMR line into two lines with equal intensity, while each X spin whose state is described by the longitudinal density element, *X*_z_, splits the NMR line into two lines with opposite intensity (Fig. 3 ). For the 2*N*_+_ ***H***_z_ coherence considered above, the NMR line is therefore first split into two lines with opposite intensity by one *X*_z_ operator and subsequently split by three identity operators, which leads to the 1:2:0:−2:−1 multiplet structure. The Appendix A gives a detailed description of using the modified Pascal’s triangle to describe the ^15^N antiphase spectra of ^15^NH_4_^+^ and Table 5 gives a complete list of expected relative intensities for the possible evolutions and detections of antiphase coherences.

**Table 5:** Expected relative intensity ratios for evolution and detection of density spin-operators of the Cartesian basis.^a^

*σ*_Evolve_	*σ*_Detect_				
	*N*_+_	2*N*_+_***H***_z_	4*N*_+_***H***_z_***H***_z_	8*N*_+_***H***_z_***H***_z_***H***_z_	16*N*_+_***H***_z_***H***_z_***H***_z_***H***_z_
*N*_+_	1:4:6:4:1	1:2:0:−2:−1	1:0:−2:0:1	1:−2:0:2:−1	1:−4:6:−4:1
2*N*_+_***H***_z_	1:2:0:−2:−1	1:1:0:1:1	1:0:0:0:−1	1:−1:0:−1:1	1:−2:0:2:−1
4*N*_+_***H***_z_***H***_z_	1:0:−2:0:1	1:0:0:0:−1	3:0:2:0:3	1:0:0:0:−1	1:0:−2:0:1
8*N*_+_***H***_z_***H***_z_***H***_z_	1:−2:0:2:−1	1:−1:0:−1:1	1:0:0:0:−1	1:1:0:1:1	1:2:0:−2:−1
16*N*_+_***H***_z_***H***_z_***H***_z_***H***_z_	1:−4:6:−4:1	1:−2:0:2:−1	1:0:−2:0:1	1:2:0:−2:−1	1:4:6:4:1

a Relative intensity ratios are calculated according to 〈exp(-iH^0t)σEvolveexp(iH^0t)|σDetect〉 followed by a separation of terms according to frequency. See Appendix A for a simple derivation based on the modified Pascal’s triangle.

It is often the case that antiphase coherences are either detected or evolved during the indirect evolution time of a 2D or 3D correlation spectrum. For example, the simplest ^15^N–^1^H HSQC correlation spectrum usually corresponds to the evolution of and indirect ‘detection’ of the singly anti-phase coherence 2*N*_x_ ***H***_z_ as described below. The operator that is indirectly detected is the operator that is transferred back to directly-detectable magnetisations, which in turn depends on the pulse sequence.

### 2.7 Application to ^15^N-ammonium bound to a protein – the 41 kDa ATP binding domain of DnaK

The equations derived above provide the basis to characterise the local dynamics and chemical exchange properties of ammonium ions in various environments. While variations of the correlation time of ammonium ions in different solvents have been measured and correlated with ammonium:solvent interactions [3], little is known about how specific monovalent cation binding sites in proteins affect the correlation time of the bound ammonium ion.

The activity of the bacterial Hsp70 homologue DnaK, an ATP-hydrolysing enzyme that functions as a molecular chaperone in the cell, relies on the binding of two potassium ions. It was shown, however, that potassium can be substituted by ammonium with the enzyme retaining more than half of its activity [38], [39]. Such enzyme-bound ^15^N ammonium ions can be observed in ^15^N edited NMR spectra in favourable cases [16], when the protein environment decreases the rate of exchange of the ammonium protons with the bulk solvent to less than ∼*J*_NH_. For the DnaK enzyme, very weak ammonium proton signals are observed in 1D ^1^H NMR spectra in the absence of nucleotide, while the addition of ADP and phosphate creates an environment that protects the ammonium ion from the bulk solvent and makes it observable in ^15^N-edited NMR spectra. The observation of ammonium NMR signals provides an opportunity for probing the properties of K^+^/NH_4_^+^ binding sites, as was shown in a previous study of the regulation of the human histone deacetylase 8 (HDAC8) by monovalent cations [16], [40]. Here we will illustrate the utility of the derived equations, taking the characterisation of K^+^/NH_4_^+^ sites a step further by probing the local correlation time of DnaK-bound ammonium from 2D ^15^N–^1^H correlation spectra.

**Fig. 4:**
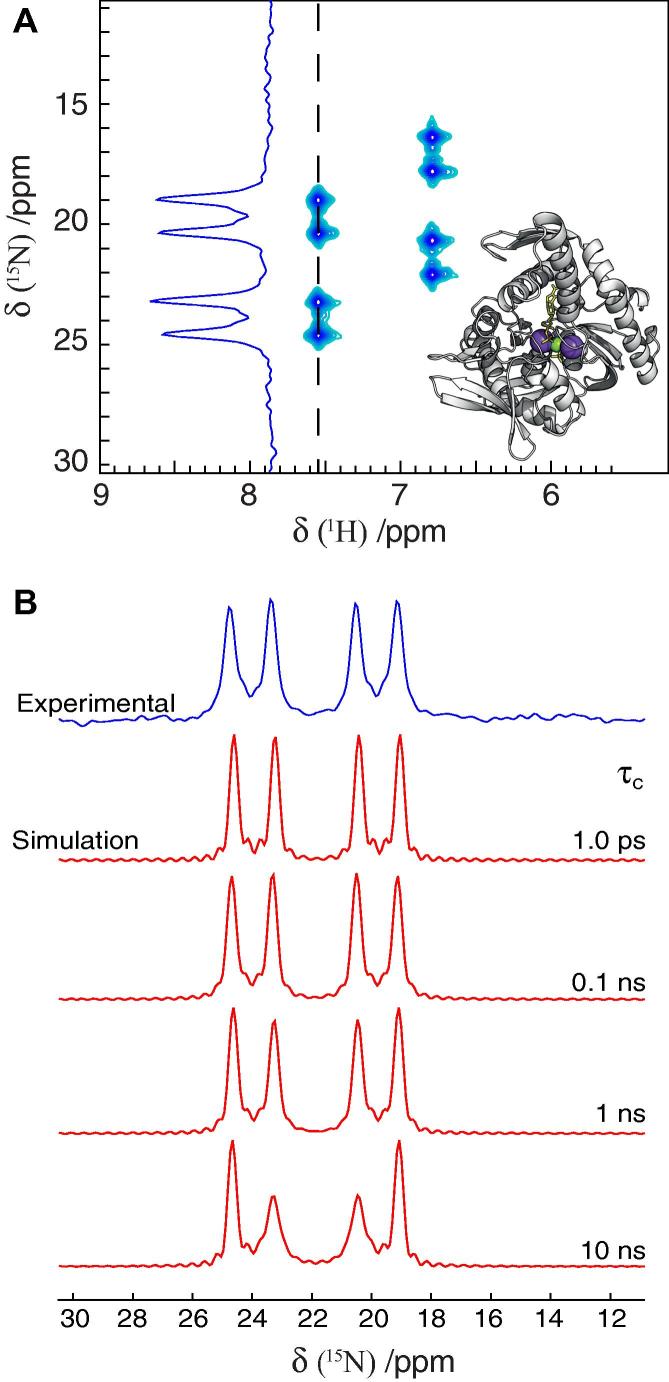
Application to ^15^N-ammonium bound to the nucleotide-binding domain of the protein DnaK. (A) ^15^N–^1^H HSQC (^1^H-coupled) correlation spectrum of ^14^N-DnaK in 150 mM ^15^NH_4_Cl (see Section 4 for full conditions). The two sets of peaks, with distinct proton chemical shifts, report individually on the two potassium binding sites of DnaK. A 1D ^15^N trace is shown for the downfield ammonium peak highlighting the relative intensities of the four observed lines at −4*πJ*_NH_, −2*πJ*_NH_, 2*πJ*_NH_, and 4*πJ*_NH_, corresponding to the transitions ν_1_, {ν_2_, ν_6_}, {ν_4_, ν_8_}, and ν_5_, respectively (inset). The crystal structure of the ATPase domain of Hsp70 (PDB: 1HPM [38]) with the two potassium ions in the active site shown as purple spheres. (B) Comparison of the experimental 1D trace of (A) with 1D ^15^N traces obtained from simulations using the equations derived above. The spectral parameters used to generate the simulated spectra are identical to those used for the experimental spectrum. *λ* = *θ* = 15 s^−1^ was assumed in the simulations, although the specific values of *λ* and *θ* do not alter the relative intensity ratio. The comparison of the experimental spectrum with a nearly 1:1:0:1:1 relative intensity ratio with the simulated spectra indicates that the local correlation time is shorter than ∼1 ns. The simulated spectra show that for slow tumbling ammonium ions, the outermost lines are significantly sharper than the inner lines due to the symmetric dipolar field created by the four protons with the same spin-state (see main text). The other ammonium signal, with a proton frequency of ∼6.8 ppm, shows the same pattern as discussed above.

Fig. 4a shows the ^1^H-coupled ^15^N–^1^H correlation spectrum of the 41 kDa ^14^N-ATP-binding domain of DnaK in 150 mM ^15^NH_4_Cl. Briefly, transverse antiphase 2*N*_+_***H***_z_ coherence is generated via an initial INEPT step, which is followed by indirect ^15^N chemical shift evolution without decoupling of the ^1^H–^15^N scalar coupling. Finally, the transverse nitrogen magnetisation is transferred back to transverse proton magnetisation via a reversed INEPT step followed by direct proton detection with ^15^N decoupling. Details are given in Section 4. Fig. 4a shows four lines corresponding to the four transitions, ν_1_, {ν_2_, ν_6_}, {ν_4_, ν_8_,}, ν_5_ (Fig. 1) with a relative intensity ratio of approximately 1:1:0:1:1, as is expected from Table 5. Also as expected, the central line is not observed because the central transitions ν_3_, ν_7_, ν_9_ are not included in the 2*N*_+_ ***H***_z_ density product operator (Table 1). The linewidths, which are directly proportional to the transverse relaxation rates, of the four transitions appear to be very similar and comparison with the simulated spectra in Fig. 4b shows that the local correlation time, *τ_c_*, of the DnaK-bound ammonium is shorter than approximately 1 ns.

## 3 Conclusions

In summary, we have developed the theoretical framework for calculating the ^15^N relaxation rates of ^15^N-ammonium. It was assumed that the geometric structure of the ammonium ion is that of a tetrahedron, which in turn means that symmetries of the energy eigenstates fall within the symmetries of the *T_d_* point group. We presented the equations that describe the transverse nitrogen relaxations of the ammonium ion in two basis sets, the Zeeman-derived basis and the Cartesian basis, as well as the relaxation rates of the longitudinal spin-density operators in the Cartesian basis. All dipole–dipole, auto- and cross-correlated relaxation mechanisms within the ammonium ion were explicitly included in the calculations and it was also shown how the relaxation of the ammonium protons caused by external spins can be taken into account.

An application of the derived equations to the study of the dynamics of enzyme-bound ammonium ions was described, where it was concluded that the local correlation time of ammonium bound to the 41 kDa domain of DnaK is less than ∼1 ns. Thus, the ammonium ion is rotating rapidly within the cation-binding site of DnaK, since the protein itself is expected to have a rotational correlation time of approximately 25 ns at 298 K. The narrow ^15^N NMR signals that were observed previously for protein-bound ammonium ions [16] can therefore be a consequence of two effects, (i) fast rotation of the ion within the protein binding sites as observed here for the enzyme DnaK or (ii) contributions from cross-correlated relaxation mechanisms originating from the high symmetry of the molecule as outlined in the previous sections.

The theoretical framework presented here provides an avenue for further investigations of free and enzyme-bound ammonium ions to elucidate the kinetic and dynamic aspects of monovalent cation binding. Combination of the derived equations with modifications of currently available NMR pulse sequences and experiments will thus shed more light on the local dynamics of ammonium ions in the binding sites of enzymes, thereby allowing more detailed characterisations of monovalent cation:enzyme interactions.

## 4 Materials and methods

### 4.1 Calculation of relaxation rates

The relaxation rates were calculated using an in-house Mathematica (Wolfram Research) script based on a strategy developed previously [41]. This script evaluates the Wigner matrix rotations and the commutator-relations involved and is available directly from the authors upon request.

### 4.2 Protein sample preparations

The NMR sample of the ATP binding domain of DnaK from *Thermus thermophilus* was prepared as explained previously [16]. The protein concentration was ∼50 μM in 100% H_2_O containing 150 mM ^15^NH_4_Cl, 0.5 mM ADP, 50 mM (NH_4_)H_2_PO_4_, 5 mM MgCl_2_, 1 mM DTT, 1 mM NaN_3_ and 75 mM Tris pH 7.5.

### 4.3 NMR experiments

The NMR experiment shown in Fig. 4 is a ^1^H-coupled ^15^N–^1^H HSQC, obtained from a standard ^15^H–^1^H HSQC by removing the 180° proton decoupling pulse during the indirect nitrogen evolution. The experiment was performed on a Bruker Avance III 500 MHz (11.7 T) spectrometer using an HCN inverse RT probe. The spectrum was recorded with 48 complex points in the indirect dimension, a sweep-width of 1000 Hz, and was processed using nmrPipe [42].

## Figures and Tables

**Fig. 5:**
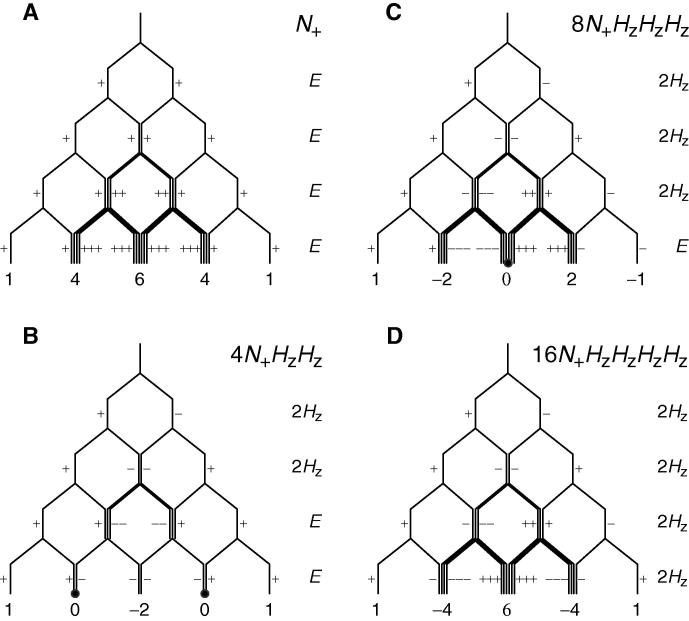
Multiplet structures of the ^15^N NMR spectrum of ^15^NH_4_^+^ calculated using the modified Pascal’s triangle described above. (A) The typical Pascal’s triangle coupling-pattern corresponding to evolution and detection of *N*_+_ resulting in a multiplet structure with relative intensities of 1:4:6:4:1. (B) A modified Pascal’s triangle depicting the situation where the 4*N*_+_***H***_z_***H***_z_ coherence is evolved and *N*_+_ is detected, which mathematically corresponds to calculating the elements 〈exp(-iH^0t)4N+HzHzexp(iH^0t)|N+〉 and separating terms according to frequency. (C and D) Show the modified Pascal’s triangles for the evolution of 8*N*_+_***H***_z_***H***_z_***H***_z_ and 16*N*_+_***H***_z_***H***_z_***H***_z_***H***_z_ with detection on *N*_+_, respectively.

## Appendix A

The strategy of using the modified Pascal’s triangle to calculate the observed multiplet structure for ^15^N coherences of the ^15^NH_4_^+^ group is described below. Fig. 5 a shows the traditional schematic representation of the Pascal’s triangle used to calculate the relative intensities of the multiplet structure of the in-phase ^15^N spectrum of ^15^NH_4_^+^. Briefly, each proton that is scalar-coupled to the ^15^N nucleus splits the NMR line into two lines with equal intensity and separated by the ^15^N–^1^H scalar coupling constant, *J*_NH_, resulting in the canonical 1:4:6:4:1 quintet structure. The intensities observed for the individual NMR lines, i.e. the population differences associated with the underlying transitions, when evolving the 2*N*_+_ ***H***_z_ coherence (and observing *N*_+_) are derived in Fig. 3. For the 2*N*_+_ ***H***_z_ coherence, the density element of one of the scalar coupled protons is described by 2*H*_z_ = (|α〉〈α| −|β〉〈β|), while the density elements of the three remaining protons are those of the identity operator *E* = (|α〉〈α| +|β〉〈β|). For the modified Pascal’s triangle, we incorporate the 2*H*_z_ density element by splitting the NMR line into two lines with opposite intensity (+) and (−). Subsequent application of three identity operators gives the relative intensities of the NMR lines obtained by evolving 2*N*_+_ ***H***_z_ and observing *N*_+_, which are 1:2:0:−2:−1 (Table 5). The relative intensities of the NMR lines and the multiplet structures that are obtained by evolving 4*N*_+_ ***H***_z_ ***H***_z_, 8*N*_+_ ***H***_z_ ***H***_z_ ***H***_z_, and 16*N*_+_ ***H***_z_ ***H***_z_ ***H***_z_ are derived in Fig. 5b–d.

Using the strategy described above, it can also be seen that evolving *N*_+_ followed by indirect ‘detection’ of 2*N*_+_ ***H***_z_, e.g. in an HSQC correlation experiment, results in a multiplet with intensity ratios of 1:2:0:−2:−1, since 〈exp(-iH^0t)σBexp(iH^0t)|σA〉=〈exp(iH^0t)σAexp(-iH^0t)|σB〉. More generally, ‘detecting’ 2*N*_+_ ***H***_z_ can in the schematic picture described here be calculated by multiplying the populations of the five lines with 1:1/2:0:−1/2:−1, which is obtained from the overlap between *N*_+_ and 2*N*_+_ ***H***_z_ for the individual transitions: that is, 1/1:2/4:0/6:−2/4:−1/1 = 1:1/2:0:−1/2:−1. Similarly, the multiplet structure obtained by indirect detection of 4*N*_+_ ***H***_z_ ***H***_z_ is calculated by multiplying the populations with 1:0:−1/3:0:1, detection of 8*N*_+_ ***H***_z_ ***H***_z_ ***H***_z_ by multiplying by 1:−1/2:0:1/2:−1, and detection of 16*N*_+_ ***H***_z_ ***H***_z_ ***H***_z_ ***H***_z_ by multiplication of 1:−1:1:−1:1. For example, evolving 4*N*_+_ ***H***_z_ ***H***_z_ followed by indirect detection of 2*N*_+_ ***H***_z_ is calculated as 1*1:0*1/2:−2*0:0*−1/2:1*−1 = 1:0:0:0:−1. The strategy outlined above is general and can also be applied to calculate AX*_n_* multiplet structures, such as AX_3_, AX_2_, and AX.
